# Echinocystic acid in *Momordica charantia L.* exosome-like nanovesicles attenuates dengue virus-induced vascular leakage associated inflammatory mediators through macrophage metabolic reprogramming and HIF-1α/p300-CBP interaction

**DOI:** 10.3389/fmed.2025.1653689

**Published:** 2025-09-30

**Authors:** Honglin Liu, Juan He, Lili Xu, Yingxuan Chen, Xiaoni Wang, Xiaoni Pang, Haihui Yang, Lianhui Liang, Shuwen Chen, Shuli Li

**Affiliations:** ^1^Department of Clinical Laboratory, Zhongshan Second People's Hospital, Zhongshan, Guangdong, China; ^2^Institute of Molecular Immunology, School of Laboratory Medicine and Biotechnology, Southern Medical University, Guangzhou, Guangdong, China

**Keywords:** *Momordica charantia L.*, echinocystic acid, dengue, macrophage, nanovesicles

## Abstract

**Introduction:**

Dengue virus (DENV) infection can progress to severe dengue hemorrhagic fever and shock syndrome, characterized by vascular leakage with high mortality. This endothelial dysfunction is primarily driven by excessive inflammatory activation of monocytes and macrophages. While *Momordica charantia L*. is known for its broad bioactive properties, its potential role in mitigating dengue-induced immunopathology remains unexplored.

**Methods:**

We investigated the effects of exosome-like nanoparticles derived from *Momordica charantia L*. (MC-ELNs) and their highly abundant constituent, echinocystic acid (EA), on DENV-induced macrophage inflammation and endothelial dysfunction. The mechanism focused on the HIF-1α–p300/CBP transcriptional complex.

**Results:**

MC-ELNs and EA significantly alleviated DENV-induced macrophage inflammation without affecting HIF-1α expression or nuclear translocation. They shifted macrophage polarization from pro-inflammatory M1 to anti-inflammatory M2 phenotype, downregulated glycolytic enzymes (HK2, PFKL, PKM1, LDHA), suppressed phagocytosis, reduced secretion of endothelial damage-associated mediators (IL-1β, IL-6, TNF-α, MMP-9), and enhanced IL-10 production. Mechanistically, both interventions inhibited the interaction between HIF-1α and p300/CBP, thereby decoupling inflammatory activation from metabolic reprogramming.

**Discussion:**

These findings reveal a novel host-directed therapeutic strategy against severe dengue by targeting the HIF-1α–p300/CBP complex. The study highlights the potential of plant-derived nanovesicles and their bioactive components, such as MC-ELNs and EA, in treating inflammatory vascular diseases.

## Graphical abstract

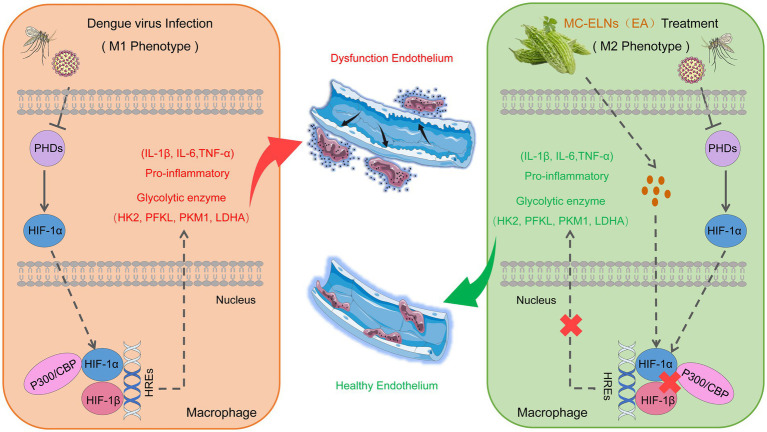

EA in MC-ELNs attenuates dengue-induced vascular leakage via HIF-1α/p300-CBP interaction and macrophage metabolic reprogramming. MC-ELNs, *Momordica charantia L*-derived exosome-like nanovesicles; EA, echinocystic acid; PHDs, prolyl hydroxylase domain-containing enzymes; p300, E1A binding protein p300; CBP, CREB-binding protein.

## Introduction

Dengue virus (DENV), a single-stranded RNA virus with four distinct serotypes (DENV 1-4) (1, 2), represents a severe global health threat (1, 2). Transmitted primarily by *Aedes aegypti* and *Aedes albopictus* mosquitoes, DENV is responsible for an estimated 390 million infections annually, endangering nearly half of the world’s population annually (3, 4). While most infections are asymptomatic or mild, a subset progresses to severe dengue hemorrhagic fever (DHF) or dengue shock syndrome (DSS), characterized by profound vascular leakage, hemorrhage, and organ failure, which contribute to significant morbidity and mortality (5, 6). Despite a global case fatality rate (CFR) of 0.1%, overwhelmed healthcare systems during outbreaks often result in higher CFRs (7, 8). Personalized fluid management hinges on timely identification of high-risk patients, yet this remains challenging during epidemics. Elucidating the molecular mechanisms and temporal dynamics of vascular leakage is thus imperative.

The pathogenesis of vascular leakage in severe dengue is multifactorial, involving: (a) direct viral effects, where immature viral particles activate Toll-like receptors (TLR-2/4) and NF-κB signaling, triggering pro-inflammatory cytokine release (8–11); (b) NS1-mediated endothelial dysfunction through cytotoxicity and induction of inflammatory mediators such as TNF-*α* and MMP-9 (12, 13); and (c) a cytokine storm [e.g., IL-1β, IL-6, TNF-α (14–16) and IL-10 (17, 18)] derived from hyperactivated immune cells including monocytes, macrophages, and dendritic cells which further disrupts endothelial integrity.

Despite its clinical significance, targeted therapies against dengue-induced immunopathology remain limited. Recent attention has turned to plant-derived exosome-like nanovesicles (ELNs) for their rich bioactive cargo and regulatory roles in inflammation (17, 19–22) and intercellular communication (23). *Momordica charantia L.*, a traditional medicinal plant, has demonstrated antidiabetic, anti-inflammatory, and antiviral activities (18, 24). Notably, its exosome-like nanoparticles (MC-ELNs) have shown protective effects in neurological and cardiotoxicity models, likely mediated through microRNA and antioxidant pathways (17). Mechanistically, miR-5266 in MC-ELNs targets the matrix metalloproteinase-9 (MMP-9) 3′-untranslated region (3’UTR) suppressing its expression and activating the AKT/GSK-3β pathway (17). Additionally, MC-ELNs ameliorate doxorubicin-induced cardiotoxicity by stabilizing p62, promoting Nrf2 nuclear translocation and HO-1 expression (25). While MC-ELNs demonstrate endothelial protective effects, their role in dengue-associated endothelial dysfunction has not been investigated.

Echinocystic acid (EA), a pentacyclic triterpenoid abundant in MC-ELNs and other herbs, exhibits broad anti-inflammatory and antioxidant properties (26, 27). It has shown efficacy in models of hypoxic–ischemic injury, arthritis, and sepsis (27–30). Nevertheless, its function in modulating macrophage-driven immunometabolic responses during viral infection and specifically in dengue remains unknown.

In our study, we demonstrate that both MC-ELNs and EA attenuate dengue-induced, macrophage-secreted inflammatory responses associated with endothelial dysfunction by specifically disrupting the HIF-1α–p300/CBP transcriptional complex. This intervention decouples inflammatory activation from metabolic reprogramming, shifting macrophage polarization toward an anti-inflammatory M2 phenotype, suppressing glycolytic flux and phagocytosis, and reducing secretion of damage-associated mediators. Our findings identify a novel host-directed strategy against severe dengue and highlight the therapeutic potential of plant-derived nanovesicles and their bioactive components in inflammatory vascular diseases.

## Results

### Isolation and characterization of MC-ELNs

**Figure 1:**
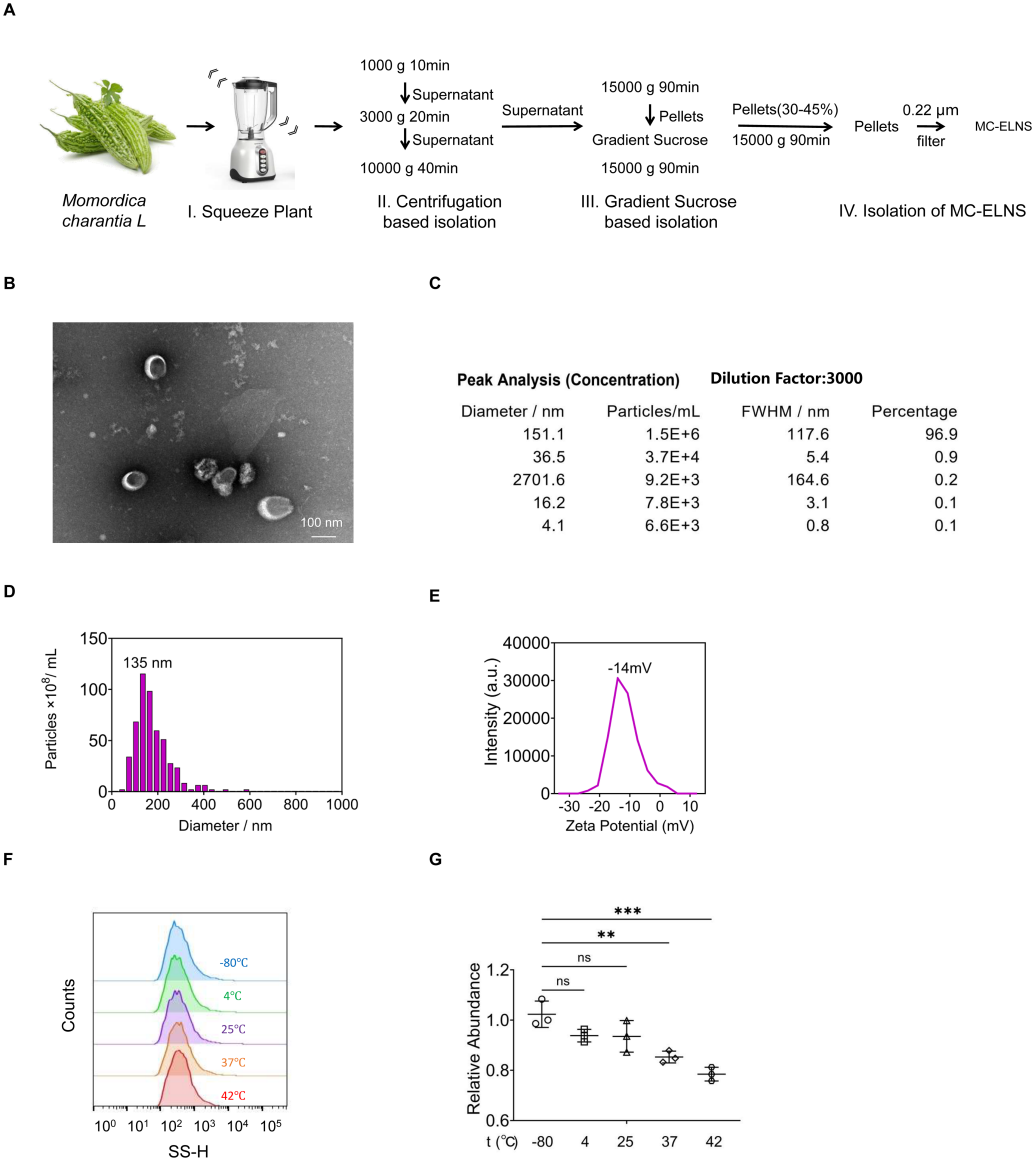
Solation and characterization of MC-ELNs. **(A)** Schematic representation of ELNs isolation from MC, MC-ELNs were purified by sucrose density gradient (8%/30%/45%/60%) under ultracentrifugation, and the band between 30 and 45% was harvested and defined as MC-ELNs; **(B)** Representative MC-ELN images obtained by Transmission Electron Microscope (TEM); **(C–E)** MC-ELN tracking analysis by Nanoparticle Tracking Analysis (NTA); **(F,G)** Stability and particle size were assessed at different temperature conditions by Flow NanoAnalyzer, **(F)** Vesicle size distribution at different temperatures; **(G)** Relative abundance of vesicles at different temperatures. A one-way ANOVA followed by multiple-comparison test **(G)** was used for statistical analysis. Data are presented as mean ± SD and are representative of at least three experiments with similar observations. ^*^p < 0.05, ^**^p < 0.01, ^***^p < 0.001, ^****^p < 0.0001. ANOVA, analysis of variance; SD, standard deviation.

To isolate intact extracellular vesicles from *Momordica charantia L*. (MC-ELNs), we employed a combination of ultracentrifugation and density gradient centrifugation (Figure 1A). Transmission electron microscopy (TEM) confirmed the presence of nanovesicles with intact bilayer membrane structures (Figure 1B). Nanoparticle tracking analysis (NTA) using a Multiple-Laser ZetaView® f-NTA system revealed a predominant subpopulation of vesicles with a diameter of 151.1 nm (96.9% of the total), and an overall mean particle size of 135 nm (Figures 1C,D). The vesicles exhibited a net negative surface charge (Figure 1E). To assess thermostability, MC-ELNs were subjected to five temperature regimens: −80 °C for 1 month, 4 °C for 1 week, 25 °C for 24 h, 37 °C for 12 h, and 42 °C for 6 h. Nanoflow cytometry (NanoFCM) analysis indicated that these conditions did not significantly affect vesicle size relative to the −80 °C control (Figure 1F). Although particle counts remained stable at 4 °C and 25 °C, incubation at 37 °C and 42 °C led to a decrease in vesicle numbers; nevertheless, relative abundance remained above 80% (Figure 1G).

### Effects of MC-ELNs on polarization and phagocytosis in DENV-2-infected BMDMs

**Figure 2:**
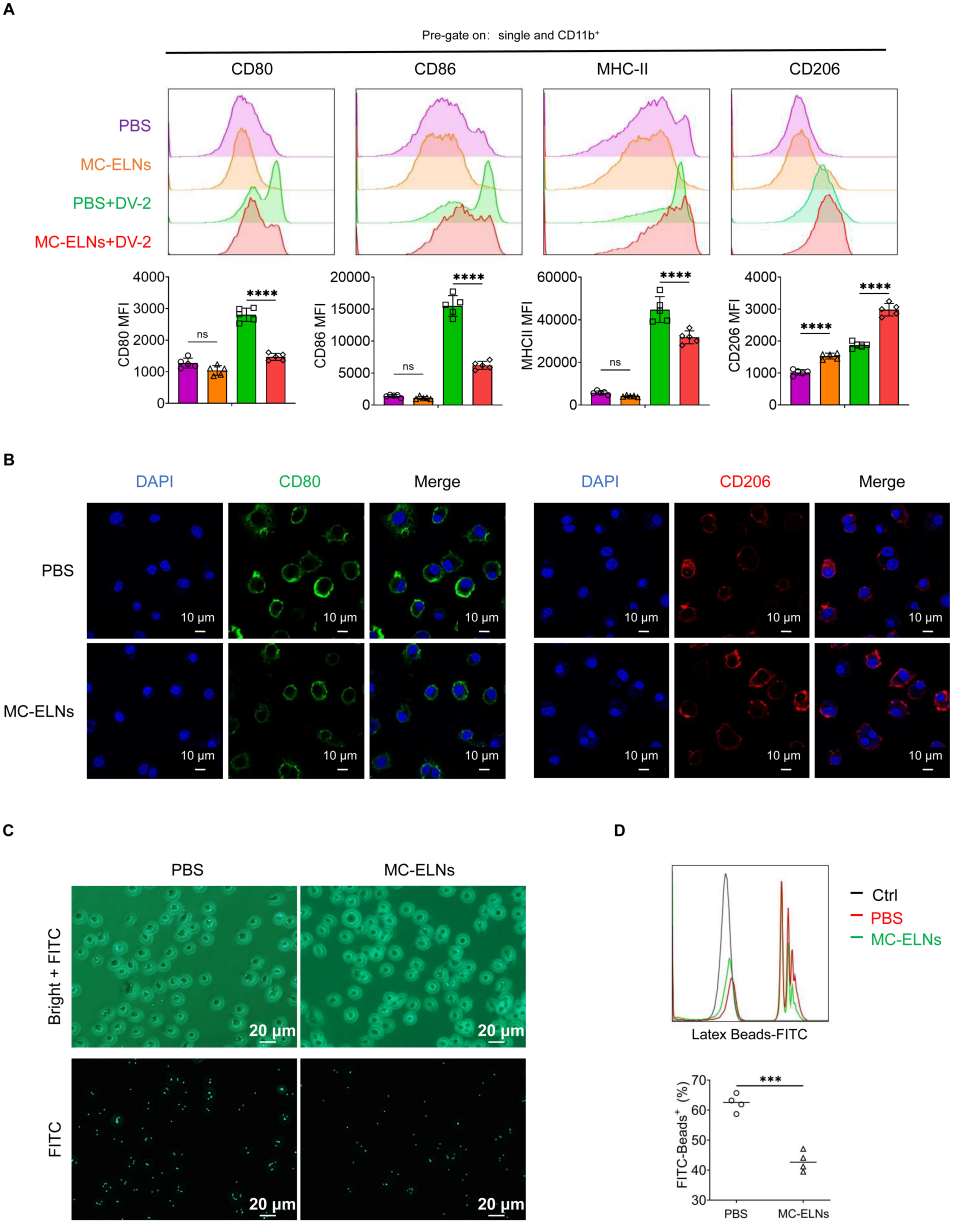
Effects of MC-ELNs on polarization and phagocytosis in DENV-2-infected BMDMs. **(A)** Flow cytometry analysis of CD80, CD86, MHC-II, and CD206 expression on DENV-2-infected BMDMs (MOI = 5) treated with MC-ELNs (10 μg/mL, 24 h; *n* = 5). **(B)** Immunofluorescence staining of CD80, CD206 on DENV-2-infected BMDMs (MOI = 5) treated with MC-ELNs (10 μg/mL, 24 h). **(C)** Fluorescent photographs of green fluorescence positive DENV-2-infected BMDMs (MOI = 5) treated with MC-ELNs (10 μg/mL, 1 h) incubated with FITC-conjugated Latex. **(D)** Flow cytometry analysis of green fluorescence-positive DENV-2-infected BMDMs (MOI = 5) treated with MC-ELNs (10 μg/mL, 1 h). incubated with FITC-conjugated latex beads (*n* = 4). A two-way ANOVA with Šidák’s *post hoc* test **(A)** and unpaired t test **(D)** was used for statistical analysis. Data are presented as mean ± SD and are representative of at least three experiments with similar observations. ^*^p < 0.05, ^**^p < 0.01, ^***^p < 0.001, ^****^p < 0.0001. ANOVA, analysis of variance; SD, standard deviation; DAPI, 4′,6-diamidino-2-phenylindole.

To evaluate whether MC-ELNs modulate immune responses in dengue virus (DENV-2 strain 16,681)-infected bone marrow-derived macrophages (BMDMs), we treated cells with MC-ELNs post-infection. Flow cytometry analysis of polarization markers (CD80, CD86, MHC-II, and CD206) revealed that MC-ELNs significantly downregulated the expression of M1 markers (CD80, CD86, and MHC-II) while upregulating the M2 marker CD206, compared to the phosphate-buffered saline (PBS) group (Figure 2A). Immunofluorescence staining of the CD80 and CD206 further supported a shift toward M2 polarization (Figure 2B), consistent with flow cytometry data. However, MC-ELNs treatment did not trigger repolarization of M2 macrophages toward the M1 phenotype (Supplementary Figure 1). To assess whether polarization changes influenced functional behavior, we measured we measured phagocytic activity using FITC-conjugated latex beads (Figure 2C). Internalization was confirmed microscopically, and flow cytometry revealed that MC-ELNs markedly suppressed phagocytosis in DENV-2-infected BMDMs (Figure 2D).

### Immunomodulatory function and metabolomic composition of MC-ELNs

**Figure 3:**
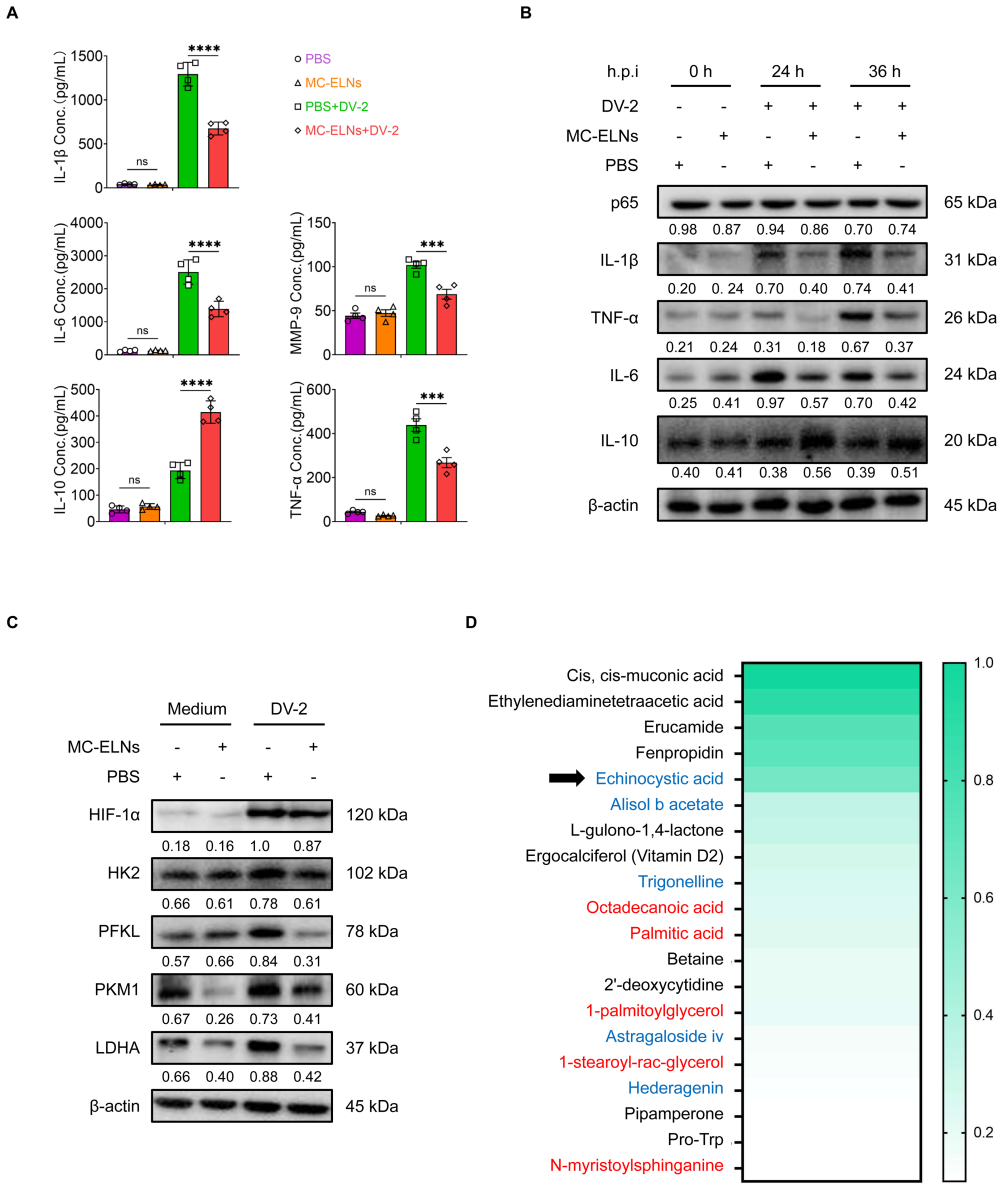
Immunomodulatory function and composition of MC-ELNs. **(A)** ELISA analysis of IL-1*β*, IL-6, IL-10, TNF-*α* and MMP-9 in DENV-2-infected BMDMs (MOI = 5) treated with MC-ELNs (10 μg/mL, 24 h; *n* = 4). **(B)** Western blot assay of the regulatory effects of MC-ELNs on Inflammatory mediators and the NF-κB core subunit p65 proteins following DENV-2 infection at MOI = 5 at 0, 24, 36 h, Brefeldin A (300 ng/mL) was added for the last 3 h of stimulation at the indicated time points. **(C)** Western blot assay of the regulatory effects of MC-ELNs on glycolysis-associated proteins following DENV-2 infection at MOI = 5 at 36 h. **(D)** Untarget metabolomics identifies the top 20 metabolites in MC-ELNs in terms of relative expression abundance, with echinocystic acid (EA) ranking fifth, the substances in red font are Structural Lipids & Membrane Components, while the substances in blue font are Natural Products & Phytochemicals. A two-way ANOVA with Šidák’s post hoc test was used for statistical analysis. Data are presented as mean ± SD and are representative of at least three experiments with similar observations. ^*^p < 0.05, ^**^p < 0.01, ^***^p < 0.001, ^****^p < 0.0001. ANOVA, analysis of variance; SD, standard deviation; hpi, hours postinfection.

Given the observed phenotypic and functional changes, we further investigated the effect of MC-ELNs on macrophage secretory profiles. Inflammatory mediators released by macrophages contribute to endothelial dysfunction, a hallmark of vascular leakage underlying dengue-related complications. Effector molecules secreted by macrophages, such as interleukin-1β (IL-1β), interleukin-6 (IL-6), interleukin-10 (IL-10), matrix metalloproteinase-9 (MMP-9), and tumor necrosis factor-alpha (TNF-*α*), have been implicated in regulating endothelial stability during dengue infection. Enzyme-linked immunosorbent assay (ELISA) of culture supernatants showed that MC-ELNs pretreatment significantly reduced levels of endothelial damage-associated mediators (IL-1β, IL-6, MMP-9, and TNF-α) and increased secretion of the protective cytokine IL-10 (Figure 3A). Western blotting corroborated these findings, demonstrating downregulation of IL-1β, IL-6, and TNF-α, and upregulation of IL-10 (Figure 3B). Notably, MC-ELNs did not alter NF-κB protein expression, a key regulator of DENV infection (Figure 3B).

Hypoxia-inducible factor-1 (HIF-1) plays a crucial role in orchestrating M1 polarization by enhancing the expression of proinflammatory genes (e.g., IL-1β), downregulating the M2 marker CD206, and inducing inducible nitric oxide synthase (iNOS) expression (31). Additionally, HIF-1 stimulates glucose uptake and the expression of key glycolytic enzymes, including pyruvate dehydrogenase kinase-1 (PDK1), thereby promoting metabolic reprogramming toward M1 polarization (32–34). Since HIF-1 is a central regulator of inflammatory polarization and glycolytic metabolism, we examined whether MC-ELNs influence HIF-1α-driven pathways. Western blot analysis revealed that MC-ELNs significantly reduced the expression of glycolysis-related proteins hexokinase 2 (HK2), phosphofructokinase liver type (PFKL), pyruvate kinase M1 (PKM1), and lactate dehydrogenase A (LDHA) without affecting HIF-1α expression (Figure 3C). These results suggest that MC-ELNs regulate macrophage glycolytic flux and polarization, as well as the secretion of endothelial damage-associated mediators, without altering HIF-1α expression during DENV infection.

To pinpoint the primary components of MC-ELNs responsible for modulating macrophage function, we performed untargeted metabolomics using UPLC-MS/MS. This analysis detected 1,065 metabolites in MC-ELNs (Supplementary Table 1), with the top 20 most abundant metabolites shown in Figure 3D. Among these, we identified several characteristic Structural Lipids & Membrane Components (Octadecanoic acid, Palmitic acid, 1-palmitoylglycerol, 1-stearoyl-rac-glycerol, N-myristoylsphinganine) as well as Natural Products & Phytochemicals (echinocystic acid, Alisol b acetate, Trigonelline, Astragaloside iv, Hederagenin). Notably, echinocystic acid (EA), a natural pentacyclic triterpenoid known for its potent antioxidant, anti-inflammatory, and analgesic properties—ranked fifth in relative abundance (Figure 3D).

### Echinocystic acid recapitulates the immunomodulatory effects of MC-ELNs

Although EA has shown efficacy in models of hypoxic–ischemic injury (28), arthritis (via suppression of Th17 cell differentiation) (30), non-small cell lung cancer (by inducing apoptosis and inhibiting migration/invasion) (29) and sepsis-associated renal inflammation and apoptosis (27). I its role in dengue-associated endothelial dysfunction remains unexplored.

**Figure 4:**
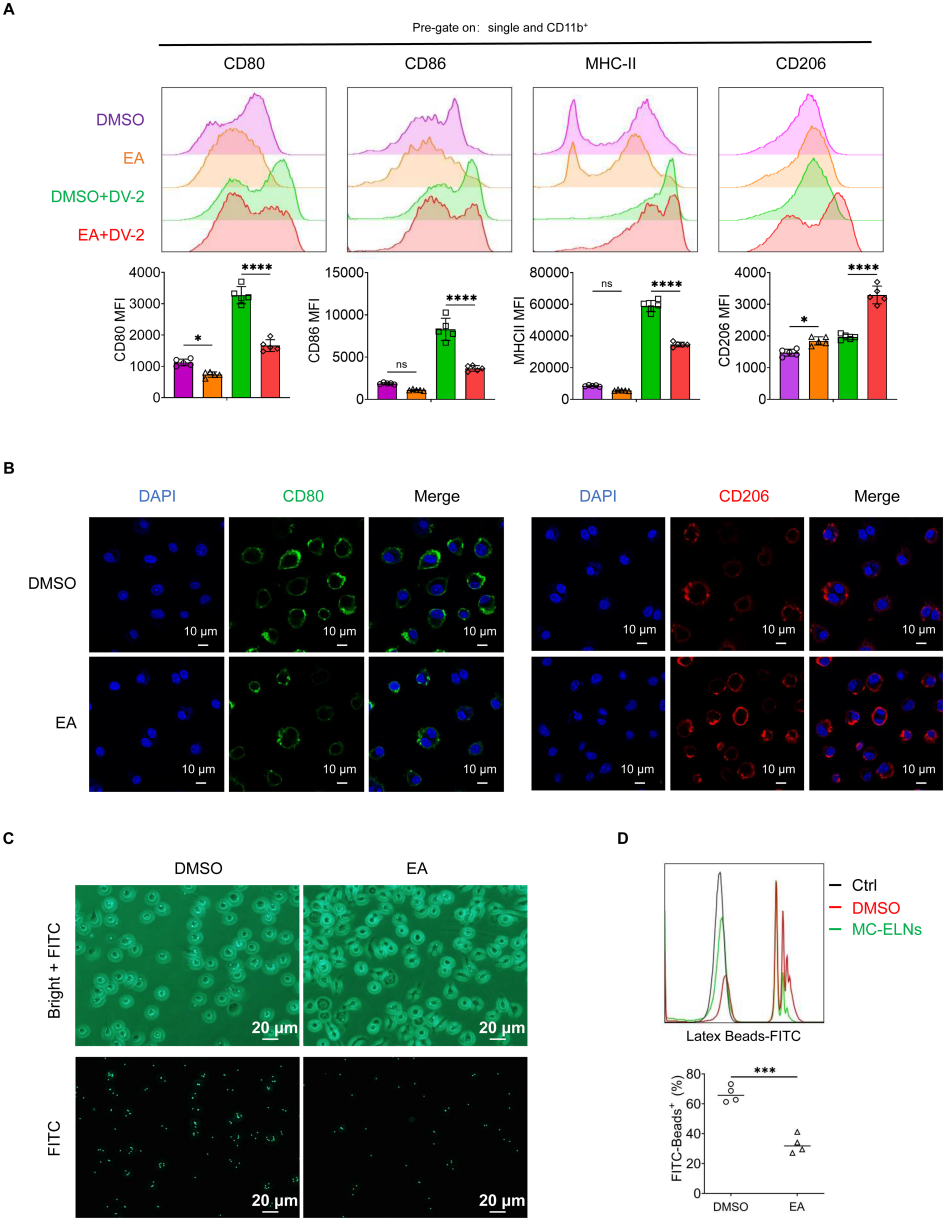
Effects of EA on polarization and phagocytosis in DENV-2-infected BMDMs. **(A)** Flow cytometry analysis of CD80, CD86, MHC-II, and CD206 expression on DENV-2-infected BMDMs (MOI = 5) treated with EA (5 μM, 24 h; *n* = 5); **(B)** Immunofluorescence staining of CD80, CD206 on DENV-2-infected BMDMs (MOI = 5) treated with EA (5 μM, 24 h). **(C)** Fluorescent photographs of green fluorescence positive DENV-2-infected BMDMs (MOI = 5) treated with EA (5 μM, 1 h) incubated with FITC-conjugated Latex. **(D)** Flow cytometry analysis of green fluorescence-positive DENV-2-infected BMDMs (MOI = 5) treated with EA (5 μM, 1 h). incubated with FITC-conjugated latex beads (*n* = 4). A two-way ANOVA with Šidák’s post hoc test **(A)** and unpaired t test **(D)** was used for statistical analysis. Data are presented as mean ± SD and are representative of at least three experiments with similar observations. ^*^p < 0.05, ^**^p < 0.01, ^***^p < 0.001, ^****^p < 0.0001. ANOVA, analysis of variance; SD, standard deviation; DAPI, 4′,6-diamidino-2-phenylindole.

To determine whether EA is a functional component of MC-ELNs, BMDMs were treated with EA after DENV-2 infection. Similar to MC-ELNs, EA downregulated M1 markers (CD80, CD86, and MHC-II) and enhanced CD206 expression (Figure 4A). Consistent with the polarization pattern observed with MC-ELNs, Immunofluorescence confirmed the promotion of an M2-like phenotype, as demonstrated by decreased CD80 and increased CD206 fluorescence signals (Figure 4B). Furthermore, EA treatment similarly suppressed phagocytosis also characteristic of MC-ELNs as evidenced in functional assays (Figures 4C,D).

### EA regulates immune responses independent of HIF-1*α* expression

**Figure 5:**
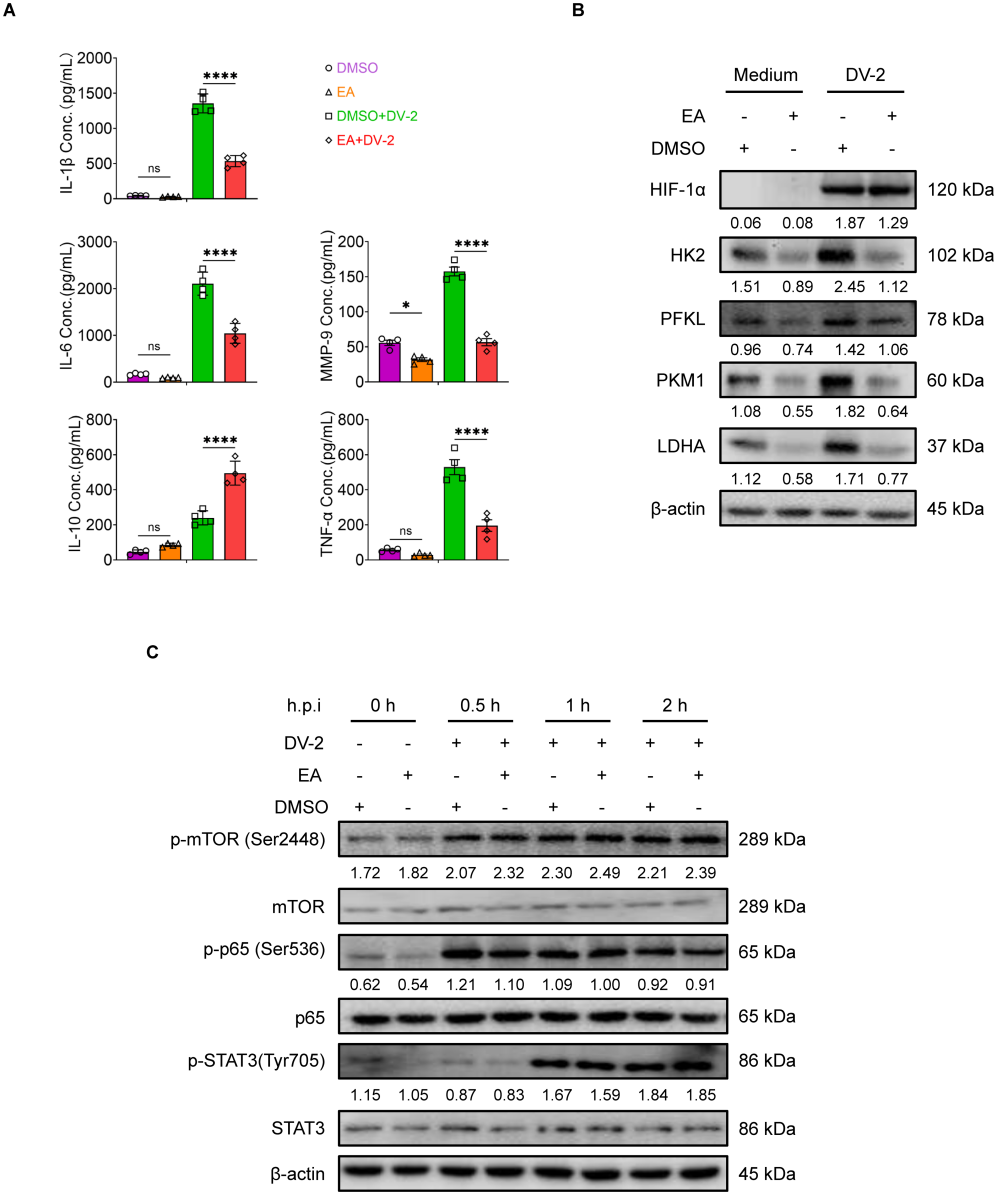
HIF-1α expression-independent immune regulation by EA in BMDMs. **(A)** ELISA analysis of IL-1β, IL-6, IL-10, TNF-α and MMP-9 in DENV-2-infected BMDMs (MOI = 5) treated with EA (5 μM, 24 h; *n* = 4). **(B)** Western blot assay of the regulatory effects of EA on glycolysis-associated proteins following DENV-2 infection at MOI = 5 at 36 h. **(C)** Western blot assay of the regulatory effects of EA (5 μM) on activation of signaling pathways following DENV-2 infection at MOI = 5 as indicated times. A two-way ANOVA with Šidák‘s post hoc test was used for statistical analysis. Data are presented as mean ± SD and are representative of at least three experiments with similar observations. ^*^p < 0.05, ^**^p < 0.01, ^***^p < 0.001, ^****^p < 0.0001. ANOVA, analysis of variance; SD, standard deviation; hpi, hours postinfection; p-, phosphorylated.

We next assessed whether EA mirrors the effects of MC-ELNs on cytokine secretion and metabolic activity. Similar to the effect observed with MC-ELNs, ELISA showed that EA reduced the release of endothelial damage-associated mediators (IL-1β, IL-6, MMP-9, and TNF-α) and elevated IL-10 production (Figure 5A). Consistent with the MC-ELNs-mediated metabolic profile, Western blot analysis indicated that EA also downregulated key glycolytic enzymes (HK2, PFKL, PKM1, and LDHA), again without altering HIF-1α expression (Figure 5B). These concordant results affirm that EA replicates MC-ELNs-mediated immunometabolic regulation of macrophage glycolytic metabolism, polarization, and endothelial-related inflammatory responses. To elucidate the mechanism of HIF-1α-independent action, we first examined upstream signaling pathways regulating HIF-1α protein and mRNA levels, including mechanistic target of rapamycin (mTOR), signal transducer and activator of transcription 3 (STAT3), and nuclear factor-kappa B (NF-κB) (35–37). Western blot analysis revealed no significant No significant changes in phosphorylation were observed following EA treatment (Figure 5C), confirming that EA does not modulate HIF-1α expression or its major regulatory pathways.

### MC-ELNs and EA inhibit HIF-1α–p300/CBP interaction

**Figure 6:**
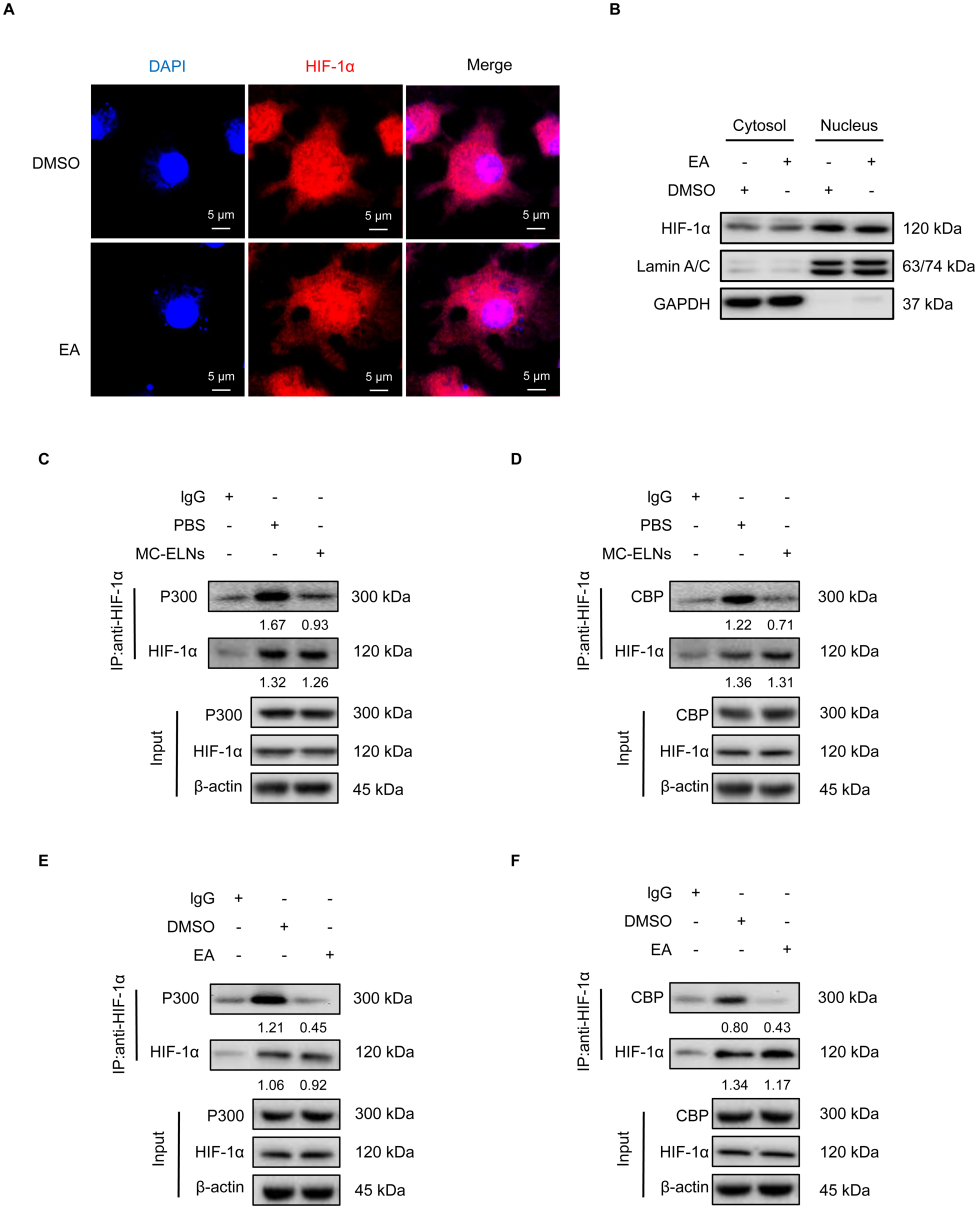
MC-ELNs/EA exert immunomodulatory effects by regulating the interaction between HIF-1α and the co-activators p300/CBP. DENV-2-infected BMDMs (MOI = 5) treated with MC-ELNs (10 μg/mL) or EA (5 μM, 4 h); **(A)** Immunofluorescence assays of HIF-1α intracellular locations in DENV-2-infected BMDMs treated with EA; **(B)** Western blotting of HIF-1α in the cytosol and nucleus fraction of DENV-2-infected BMDMs treated with EA; Lamin A/C and GAPDH were used as nuclear and cytoplasmic protein markers, respectively; **(C,D)** Co-immunoprecipitation (Co-IP) assays showed that MC-ELNs attenuated the interaction between HIF-1α and the co-activator p300 **(C)** and CBP **(D)**; **(E,F)** Co-immunoprecipitation (Co-IP) assays showed that EA attenuated the interaction between HIF-1α and the co-activator p300 **(E)** and CBP **(F)**; Protein–protein interactions were immunodetected using HIF-1α antibodies, protein extracts (input) were immunoprecipitated with HIF-1α, p300, CBP or β-actin antibody and resolved by SDS-PAGE. DAPI, 4′,6-diamidino-2-phenylindole.

Under hypoxia, HIF-1α accumulates and translocate to the nucleus to dimerize with HIF-1β and recruit coactivators p300/CBP (36). HIF-1 then binds to hypoxia-response elements (HREs) in target gene promoters to initiate transcription (36). Although immunofluorescence (Figure 6A) and cellular fractionation (Figure 6B) showed that EA did not affect HIF-1α nuclear translocation, co-immunoprecipitation assays revealed that both MC-ELNs and EA significantly disrupted the interaction between HIF-1α and p300/CBP in infected BMDMs (Figures 6C–F). These results demonstrate that EA, from MC-ELNs attenuates the association between HIF-1α and the transcriptional coactivators p300/CBP in DENV-2-infected BMDMs. This disruption leads to suppressed glycolytic flux, attenuated M1 polarization, and downregulation of inflammatory mediators associated with endothelial injury, collectively contributing to the alleviation of vascular leakage.

## Discussion

Dengue hemorrhagic fever (DHF) and dengue shock syndrome (DSS), characterized by severe vascular leakage and systemic inflammation, remain critical global health challenges with limited therapeutic options. In our study, we demonstrate that extracellular vesicles derived from *Momordica charantia L*. (MC-ELNs) and their predominant bioactive metabolite, echinocystic acid (EA), significantly modulate macrophage immune responses during DENV-2 infection, thereby attenuating endothelial injury and potentially mitigating vascular leakage—a hallmark of severe dengue (Graphic Abstract).

Building upon previously reported neuroprotective and cardioprotective properties of MC-ELNs (17, 25), our study reveals a novel immunomodulatory role in dengue infection. We demonstrate that MC-ELNs drive a shift in macrophage polarization from a pro-inflammatory M1 phenotype toward an anti-inflammatory M2 state, as evidenced by downregulation of CD80, CD86, and MHC-II, and concomitant upregulation of CD206. This repolarization was accompanied by functional changes, including suppressed phagocytic activity and altered cytokine secretion, with reduced levels of IL-1β, IL-6, MMP-9, and TNF-*α* and enhanced IL-10 production. This dual regulatory effect positions MC-ELNs as a promising strategy to counteract the cytokine storm-driven vascular pathology in severe dengue.

Notably, these immunomodulatory effects occurred independently of changes in HIF-1α expression, its upstream regulatory pathways such as mTOR, STAT3, and NF-κB, or its intracellular distribution. Instead, both MC-ELNs and EA disrupted the interaction between HIF-1α and its transcriptional coactivators p300/CBP, leading to inhibition of glycolytic metabolism and suppression of M1-polarizing.

Metabolomic profiling identified EA as a primary constituent of MC-ELNs, and functional assays confirmed that EA recapitulates the immunometabolic effects of the parent vesicles. Although EA ranked fifth in abundance among the detected metabolites, the four preceding compounds were identified as either LC–MS reagent artifacts (e.g., cis,cis-muconic acid and ethylenediaminetetraacetic acid) or residual agrochemicals such as fenpropidin. Enzalutamide, a second-generation nonsteroidal antiandrogen used primarily for the treatment of castration-resistant prostate cancer (CRPC) (38), has no previously reported role in inflammation modulation. While the anti-inflammatory and anti-apoptotic properties of EA have been described in various disease contexts (27–30), its function in viral infections particularly its immunometabolic regulatory effects has remained unexplored. In our study, EA similarly suppressed glycolysis, promoted M2 polarization, inhibited phagocytosis, and reduced inflammatory mediator release, all without affecting HIF-1α nuclear translocation or expression.

The discovery of EA’s endothelial-protective effects opens avenues for adjunctive therapies in dengue management. By tempering macrophage-driven inflammation and metabolic dysregulation, EA could complement existing supportive care to reduce complications like plasma leakage and shock. However, several limitations warrant attention: (a) Cell-type specificity: Whether MC-ELNs/EA exert direct effects on endothelial cells or other immune populations (e.g., dendritic cells or T cells) remains untested; (b) *In vivo* validation: The therapeutic efficacy and pharmacokinetics of EA require confirmation in animal models of dengue; (c) Broader pathophysiological relevance: Given HIF-1α’s role in tumor progression, the safety of long-term EA administration in cancer-prone individuals needs evaluation; (d) Translational gaps: Clinical studies are essential to determine optimal dosing regimens and potential synergies with antiviral agents.

These findings collectively demonstrate that MC-ELNs and EA confer protection not through canonical hypoxia-signaling pathways, but rather via targeted disruption of the HIF-1α–p300/CBP complex. This intervention effectively decouples inflammatory activation from metabolic reprogramming in macrophages, revealing a novel therapeutic strategy against dengue-associated immunopathology with potential relevance to other inflammatory vascular disorders. Our study repositions *Momordica charantia L*. not only as a nutraceutical source but also as a producer of biologically active nanovesicles (MC-ELNs) with multi-faceted therapeutic properties. By identifying EA as the key effector behind MC-ELNs’ immunometabolic actions and elucidating its role in attenuating the interaction between HIF-1α and the transcriptional coactivators p300/CBP, we establish a mechanistic basis for therapeutic intervention. These insights enhance our understanding of host-targeted antiviral approaches and highlight the value of plant-derived exosome-like nanoparticles in integrating traditional medicine with contemporary therapeutics. Subsequent research incorporating multi-omics analyses and preclinical models will further accelerate the translation of MC-ELNs/EA toward clinical applications.

## Materials and methods

### Cells and agents

*Aedes albopictus* mosquito C6/36 (C6/36, CRL-1660, ATCC®) cells were grown and maintained in MEM supplemented with 1 mM sodium pyruvate, 1%, 100 × non-essential amino acid, 2mML-glutamin, and 10% FCS (both from Gibco™, Life Technology), at 28 °C with 5% CO2. Femurs of 6 to 8-week female AG129 mice (average weight: 20 g) were collected and incubated in DMEM medium (Corning Inc., Manassas, VA, United States) containing 40 ng/mL murine M-CSF (PeproTech, Rocky Hill, NJ, United States), with medium replacement every 2 days, and on day 5, to obtain primary BMDMs. BMDMs were treated with the following agents as indicated in the figure legends: echinocystic acid (EA, 5 μM, MedChemExpress LLC.); MC-ELNs (10 μg/mL).

### Mice

SPF AG129 mice were provided by the Southern Medical University Laboratory Animal Management Centre of Southern Medicine University (Guangzhou, China). The experiment protocol was approved by the Biosafety Management Committee and the Medical Ethics Committee of Zhongshan Second people’s Hospital (NO.2024058), all experiments were performed in accordance with relevant guidelines and regulations. Studies involving mice to be reported as described by the ARRIVE guidelines.

### DENV-216681 preparation of and infection

DENV-2 strain 16,681 was originally obtained from ATCC and propagated in 80% confluent C6/36 cells cultured in MEM supplemented with 1 mM sodium pyruvate, 1% 100 × non-essential amino acids, 2 mM L-glutamine, and 10% FCS (all reagents from Gibco™, Life Technologies). On day 7, the medium was refreshed, and on day 9, the supernatant was harvested and cleared from cellular debris by centrifugation and subsequent filtration using a 0.2 mM filter. Viral aliquots were snap frozen in liquid nitrogen and stored at 80 °C, viral titers were determined as described by Lambeth et al. (39).

BMDM were infected with DENV-2 on the basis of infectious virus particles per milliliter as indicated in the figure legends, the supernatants were stored at −80 °C for ELISA assays (Multi Sciences, Lianke Biotech), cells were collected for flow cytometry analysis or lysed for western blot analysis.

### Isolation and characterization of *Momordica charantia L.*-exosome-like nanoparticles

MC-ELNs were isolated by continuous differential centrifugation, ultracentrifugation, and then purified by gradient sucrose solution as described previously (17). Briefly, *Momordica charantia L*. was squeezed and continuously centrifuged at 1,000 g for 10 min, 3,000 g for 20 min, and 10,000 g for 40 min at 4 °C. The obtained supernatant was ultracentrifuged at 150,000 × g for 90 min (Beckman Optima L-100XP, Beckman, United States), and the pellets were resuspended in phosphate-buffered saline (PBS), transferred to a gradient sucrose solution (8, 30, 45, and 60%), and ultracentrifuged at 150,000 × g for another 90 min. The band between the 30 and 45% sucrose layer was collected and ultracentrifuged at 150,000 × g for 90 min and passed through a 0.22 μm filter to obtain sterile MC-ELNs. The protein concentration of MC-ELNs was evaluated by the BCA assay kit (Beyotime, P0011).

For transmission electron microscope imaging, MC-ELNs were adsorbed on a carbon-coated grid. The grid was stained with 1% uranyl acetate. The absorbed MC-ELNs were examined under a Tecnai G2 Spirit Twin Transmission Electron Microscope (TEM), and the images were recorded with an AMT 2 k CCD camera. The size distribution of the MC-ELNs was analyzed by using Multiple-Laser ZetaView® f-NTA Nanoparticle Tracking Analyzers (Particle Metrix, Germany).

To evaluate stability under different temperature conditions, the vesicles were incubated under five different regimes: −80 °C for 1 month, 4 °C for 1 week, 25 °C for 24 h, 37 °C for 12 h, and 42 °C for 6 h. Their stability and particle size were assessed using Flow NanoAnalyzer (NanoFCM, Xiamen Flow Biotechnology Co., Ltd.).

### Flow cytometry

BMDMs were seeded at a density of 4 × 10^5^ cells per well, followed by infection with DENV-2 (MOI = 5) and treatment with either MC-ELNs or EA for 12 h. These cells were digested with EDTA-free trypsin, incubated in 1% FBS-PBS containing the following antibodies at 4 °C in dark for 30 min: FITC-CD80 (16-10A1; Thermo Fisher), APC-CD86 (GL-1; Tonbo), PE-Cy7-MHC-II (M5/114.15.2; Invitrogen), eFluor 450-CD11b (M1/70; Tonbo) and PE-CD206 (C068C2; BioLegend).

To assess the phagocytic capacity of DENV-2-infected macrophages under different treatment conditions (PBS vs. MC-ELNs or DMSO vs. EA), BMDMs were seeded at a density of 4 × 10^5^ cells per well, followed by infection with DENV-2 (MOI = 5) and treatment with either MC-ELNs or EA for 1 h. The cells were incubated with FITC-conjugated latex beads (Cat#: L4530, Merck Millipore). Following confirmation of bead internalization by fluorescence microscopy, the cells were harvested for subsequent analysis.

Cells were then centrifuged at 300 g for 5 min, fixed with 4% paraformaldehyde, and then were detected using Attune NxT flow cytometry (Thermo Fisher) and the data were analyzed with the Flowjo 10 software (BD Biosciences).

### Enzyme-linked immuno-assay

BMDMs were plated at a density of 4 × 10^5^ cells per well, followed by infection with DENV-2 (MOI = 5) and treatment with either MC-ELNs or EA for 12 h. The supernatants were stored at −80 °C for ELISA assays (Multi Sciences (Lianke) Biotech, Co., Ltd. Hangzhou, Zhejiang, China) and detection the concentration of IL-1β、IL-6, IL-10, MMP-9, TNF-*α* according to the instructions of the producers.

### Western blot and co-IP assay

To detect inflammatory mediators, BMDMs were seeded in 12-well plates at a density of 4 × 10^5^ cells per well, infected with DENV-2 at an MOI of 5, and treated with MC-ELNs. Brefeldin A (300 ng/mL, HY-16592, MCE) was added for the last 3 h of stimulation at the indicated time points (0, 24, and 36 h). For analysis of the NF-κB core subunit p65, BMDMs were plated at 4 × 10^5^ cells per well, infected with DENV-2 (MOI = 5), and treated with MC-ELNs at 0, 24, and 36 h.

To assess glycolysis-related proteins, BMDMs were seeded at 4 × 10^5^ cells per well, infected with DENV-2 (MOI = 5), and treated with either MC-ELNs or EA for 36 h. For detection of signaling pathway proteins, BMDMs were plated at 4 × 10^5^ cells per well, infected with DENV-2 (MOI = 5), and treated with EA for 0, 0.5, 1, and 2 h.

BMDMs were harvested and lysed with Western & IP cell lysis buffer (Beyotime, P0013) containing 1 mg/mL protease inhibitor cocktail (Sigma-Aldrich, P8340) for 30 min on ice. After centrifugation for 30 min at 12,000 g at 4 °C, the protein concentration in the supernatant of each sample was determined using a BCA protein assay kit (Beyotime, P0011). Twenty micrograms of protein from each samples were run on 10% SDS-PAGE (Solarbio, S8010) and transferred to PVDF membranes (Millipore, K2MA8350E) after electrophoresis. The membranes were applied to 1 × TBST (Solarbio, 71080) and 5% skimmed milk (BD, 2271470), using following primary antibodies with the dilution of 1:1000: anti-IL-1*β* (26048-1-AP, Proteintech), anti-IL-6 (83795-7-RR, Proteintech), anti-IL-10 (82191-3-RR, Proteintech), anti-TNF-*α* (17590-1-AP, Proteintech), anti-HIF-1α (#36169), anti-hexokinase 2 (HK2, #2867), anti–phosphofructokinase liver type (PFKL, #8175), anti–pyruvate kinase M1 (PKM1, #7067), and anti–lactate dehydrogenase A (LDHA, #2012), mTOR (#2972), p-mTOR (Ser2448) (#2971), NF-κB p65 (#8242), p-NF-κB p65 (Ser536) (#3033), Stat3 (#4904), p-Stat3 (Tyr705) (#9145), (Cell Signaling Technology, Inc., Beverly, MA, United States); anti-β-Actin (1:5000; #HC201, Transgen Biotech.) and the corresponding HRP-conjugated secondary antibodies (1:5000; Goat anti-Mouse IgG (H + L) Cat#: 31430, Goat anti-Rabbit IgG F(ab’)2 Cat#: 31234, ThermoFisher) overnight at 4 °C, washed three times. The signals were developed using FDbio-Pico ECL (Hangzhou Fude Biological Technology Co., Ltd., Hangzhou, Zhejiang, China) and obtained using FluorChem (ProteinSimple, Wallingford, CT, United States).

For the co-IP assays, the precleared supernatants of cell lysates were incubated with anti-HIF-1α antibody plus protein A/G magnetic beads overnight at 4 °C with slow rotation. The formed immunoprecipitation pellets were washed five times with Tris-buffered saline (Solarbio, T1150) containing 0.1% Tween 20 with the aid of a magnetic shelf (MCE), and then subjected to western blot analyses.

### Untargeted metabolomic analysis

Extracted and purified exosome-like vesicles from *Momordica charantia L*. were sent to six duplicate samples extracted from different batches for Untargeted metabolomics analysis, Untarget global metabolomic profiles were generated through Metabolon (Research Triangle, NC) using ultraperformance liquid chromatography coupled with high-resolution/accurate mass spectrometry (UPLC-MS/MS). Two platforms were used to detect a comprehensive list of metabolites: (1) UPLC-MS/MS under positive ionization, (2) UPLC-MS/MS under negative ionization. Metabolites were identified by their m/z retention time and through comparison with library entities of purified known standards.

### Subcellular fraction

BMDMs were seeded at a density of 1 × 10^7^cells per plates followed by infection with DENV-2 (MOI = 5) and treatment with EA for 4 h. To detect the intracellular localization of HIF-1α (#36169, CST), subcellular fraction of BMDMs was performed using the Nuclear-Cytosol Extraction Kit (Applygen Technologies Inc., Beijing, China) as per the instruction of the manufacturer. The obtained cellular fractions of protein were analyzed using western blotting, with Lamin A/C (#4777, CST) and GAPDH (10494-1-AP, Proteintech) as the reference proteins of nucleus fractions and cytosol, respectively.

### Immunofluorescence staining and confocal microscopy observation

BMDMs were seeded on glass coverslips in 12-well plates at a density of 1 × 10^4^ cells per well in 500 μL DMEM. Following adhesion, cells were infected with DENV-2 at an MOI of 5 and subsequently treated with MC-ELNs or EA for 24 h. The expression of M1 marker CD80 and M2 marker CD206 was assessed by immunofluorescence staining using anti-CD80 antibody (1:200, #66406-1-Ig, Proteintech) and anti-CD206 antibody (1:200, #83485-1-RR, Proteintech), respectively.

In a parallel experiment, BMDMs were similarly seeded, infected with DENV-2 (MOI = 5), and then treated with EA for 4 h to evaluate early effects. Intracellular HIF-1α levels were detected using immunofluorescence staining with an anti-HIF-1α antibody (1:200, #36169, CST).

Cells were then counterstained with DAPI and observed with Zeiss Axiovert LSM880 confocal laser scanning microscope (Carl Zeiss Microscopy, LLC., Thornwood, NY, United States).

### Statistical analysis

The representative data of at least three independent experiments were presented as means ± standard deviation (s.d.). *t* test was used to compare the difference when two parameters were involved. The difference of a response variable affected by more than three (inclusive) parameters of one factor was compared using one-way ANOVA. Two-way ANOVA was used to analyze the influence of two independent factors on a response variable and determine the existence of interaction between the two factors on this response variable. Least Significant Difference (LSD) or Dunnett’s T3 method was used for *Post hoc* multiple comparisons. *p* < 0.05 indicated that the difference in treatment groups was statistically significant. All statistical analyses were performed using GraphPad Prism 9.4.1 (San Diego, CA).

## Data Availability

The original contributions presented in the study are included in the article/Supplementary material, further inquiries can be directed to the corresponding authors.
